# Clinical impact of the BIOFIRE FILMARRAY Pneumonia Panel combined with antimicrobial stewardship education on pediatric patients with lower respiratory tract infection: a pre–post study from a tertiary center in Nara, Japan

**DOI:** 10.1128/spectrum.02213-26

**Published:** 2026-09-22

**Authors:** Daisuke Kitagawa, Antonio Di Lorenzo, Takumi Okamura, Soshi Hachisuka, Naohiro Yamamoto, Hiroki Nishikawa, Masayuki Onaka, Rika Suzuki, Soma Suzuki, Fumihiko Nakamura, Sayaka Yoshida, Taito Kitano

**Affiliations:** 1 Department of Laboratory Medicine, Nara Prefecture General Medical Center 36927, Nara, Japan; 2 Clinical Research Center, Nara Prefecture General Medical Center 36927, Nara, Japan; 3 Department of Pediatrics, Nara Prefecture General Medical Center 36927, Nara, Japan; 4 Interdisciplinary Department of Medicine, Aldo Moro Bari University, Bari, Italy; Icahn School of Medicine at Mount Sinai, New York, New York, USA

**Keywords:** BIOFIRE FILMARRAY Pneumonia Panel, FAPN, pediatric pneumonia, antimicrobial stewardship, multiplex PCR, lower respiratory tract infection, watch antibiotics, interrupted time-series

## Abstract

Lower respiratory tract infections (LRTIs) account for a large share of pediatric antimicrobial prescriptions. The BIOFIRE FILMARRAY Pneumonia Panel (FAPN; BioMérieux) detects 33 respiratory pathogens within approximately 1 h. We investigated the clinical impact of its systematic introduction—combined with antimicrobial stewardship education. A retrospective cohort study was conducted at a tertiary pediatric center in Japan, enrolling all children aged <18 years with a lower respiratory tract infection between April 2023 and April 2026. In the post-FAPN period (April 2025–April 2026), the FAPN was implemented, alongside structured stewardship education incorporating a clinical decision flowchart. Logistic regression and generalized linear model (GLM) regression with log-link negative binomial distribution were employed for categorical and quantitative outcomes, respectively. Interrupted time-series analysis (ITSA) was performed using bimesters as time units and Newey-West standard errors. A total of 815 children were enrolled (pre-FAPN: 590; post-FAPN: 225). Antimicrobial prescribing rates were significantly lower following FAPN implementation (adjusted odds ratio, aOR: 0.39; 95% CI: 0.28–0.55; *P* < 0.001). Adjusted GLM regression identified a significant reduction in broad-spectrum antibiotic DOTs (adjusted β: −0.41; 95% CI: −0.66 to −0.05; *P* = 0.001), while narrow-spectrum antibiotic DOTs were unchanged. ITSA also confirmed a significant reduction in antimicrobial prescription rate (β: −0.53; 95% CI: −0.84 to −0.21; *P* = 0.001). The FAPN, combined with structured stewardship education, was significantly associated with reduced antimicrobial prescribing and a shift away from broader-spectrum antimicrobials. These findings support the clinical value of rapid multiplex PCR diagnostics in pediatric LRTIs when integrated with a strong antimicrobial stewardship program.

**IMPORTANCE:**

A strong stewardship education program may need to be combined with the introduction of a multiplex PCR test for pediatric pneumonia. With a clear decision flowchart, clinicians could successfully reduce antimicrobial use and shift their antimicrobial prescriptions to narrower ones.

## INTRODUCTION

Lower respiratory tract infections (LRTIs), including community-acquired pneumonia (CAP), represent one of the most frequent causes of pediatric hospitalization and antimicrobial prescription worldwide (1–3). In Japan, pediatric pneumonia accounts for a substantial proportion of acute admissions, driven by a broad spectrum of causative pathogens ranging from respiratory viruses—such as respiratory syncytial virus (RSV), human rhinovirus/enterovirus (HRV/EV), and human metapneumovirus (hMPV)—to bacterial pathogens including *Streptococcus pneumoniae*, *Haemophilus influenzae*, *Mycoplasma pneumoniae*, and *Moraxella catarrhalis* (4). The co-existence of viral and bacterial pathogens in pediatric LRTIs complicates clinical decision-making and may inadvertently drive unnecessary antibiotic use (5).

Antimicrobial stewardship programs (ASPs) in pediatric settings aim to reduce unnecessary antibiotic prescriptions and mitigate antimicrobial resistance (AMR). The World Health Organization (WHO) AWaRe (Access, Watch, Reserve) classification was introduced to guide antimicrobial prescribing globally (6). “Access” antibiotics (e.g., amoxicillin, first-generation cephalosporins) are recommended as first-line agents for common infections, whereas “Watch” antibiotics (e.g., third-generation cephalosporins, carbapenems) carry a higher risk of resistance selection and should be reserved for specific indications (6, 7). Monitoring the balance between “Access” and “Watch” antibiotic use is increasingly recognized as a meaningful stewardship metric (8).

Traditional microbiological diagnosis of LRTIs relies on cultures and targeted antigen testing, both of which have significant limitations in sensitivity, speed, or pathogen coverage (9, 10). The BIOFIRE FILMARRAY Respiratory Panel 2.1 (FARP; BioMérieux) simultaneously detects up to 22 viral and bacterial targets within approximately 1 h (11). However, it lacks semi-quantitative bacterial load data, resistance gene detection, and coverage of key bacterial pneumonia pathogens such as *S. pneumoniae* and *H. influenzae*.

The BIOFIRE FILMARRAY Pneumonia Panel (FAPN; BioMérieux, Marcy-l'Étoile, France) addresses these limitations by detecting 33 targets—including detection of 18 bacteria with semi-quantitative load reporting for 15 bacteria, eight viruses, and seven antimicrobial resistance genes (*mecA/C*, *CTX-M*, *KPC*, *NDM*, *OXA-48-like*, *IMP*, *VIM*)—within approximately 60 min (12, 13). Studies in adult ICU and general ward settings have demonstrated its diagnostic performance and stewardship impact (14–16); a pre–post study in a Japanese pediatric intensive care unit (PICU) also identified improved pathogen identification following multiplex PCR implementation, although its impact on antimicrobial use was limited without an accompanying stewardship program (17). Evidence on the combined effect of rapid multiplex testing with structured antimicrobial stewardship education in a general pediatric ward and its impact on AWaRe-classified prescribing remains limited.

At our center, the FAPN was systematically introduced on 14 April 2025. Concurrently, all pediatricians received structured antimicrobial stewardship education, including a clinical decision flowchart guiding antibiotic selection based on FAPN results and clinical severity. This study aims to evaluate the clinical impact of this dual intervention on: (i) antimicrobial prescribing rates; (ii) total days of therapy (DOTs) in treated patients, with a focus on AWaRe classification; (iii) rapid pathogen identification rates; and (iv) length of hospital stay (LOS). Hospitalization rate was not included as a primary outcome because the FAPN is performed almost exclusively in patients for whom a decision regarding admission has already been made; the rate therefore reflects clinical severity at presentation rather than the diagnostic panel’s impact.

## MATERIALS AND METHODS

### Study design and settings

This was a population-based, retrospective cohort study with a pre–post design, conducted at Nara Prefecture General Medical Center (Nara, Japan), a tertiary referral hospital with dedicated pediatric, neonatal intensive care, surgical, and general intensive care units. The study protocol was approved by the Institutional Review Board of Nara Prefecture General Medical Center (approval number: 960). Owing to the retrospective observational design, the requirement for written informed consent was waived. The study was performed in full compliance with the Declaration of Helsinki.

### Study population

Eligible study participants were all children aged under 18 years diagnosed with LRTI among hospitalized cases or pneumonia among outpatient cases at Nara Prefecture General Medical Center between April 2023 and April 2026. Throughout the study period, LRTI and pneumonia were clinically diagnosed by pediatricians. LRTI was based on acute cough plus other lower respiratory tract symptoms, including wheeze, rhonchi, and difficulty breathing, and pneumonia was clinically diagnosed based on the findings of LRTI plus abnormalities in physical findings (i.e., crackles, dullness of percussion, and decreased breath sounds) or chest X-ray findings (18, 19). The rationale for excluding outpatient LRTI cases without pneumonia was the expected lack of benefits of FAPN, as well as the diagnostic stewardship perspective given the very low antimicrobial prescription cases in this population. Diagnoses were established by the attending pediatrician based on clinical criteria (fever, cough, tachypnea, and/or oxygen desaturation) with or without radiographic confirmation.

### Study periods and intervention

#### Pre-FAPN period (April 2023–13 April 2025)

The FARP was employed as the primary rapid multiplex test, detecting up to 22 viral and bacterial/atypical targets, including RSV, influenza A/B, HRV/EV, adenovirus, hMPV, parainfluenza virus types 1–4, coronavirus subtypes, SARS-CoV-2, *Mycoplasma pneumoniae*, *Chlamydophila pneumoniae*, *Bordetella pertussis*, and *Bordetella parapertussis*. Only nasopharyngeal swabs were used for FARP.

#### Post-FAPN period (14 April 2025–April 2026)

The FAPN was implemented as the standard diagnostic tool using sputum samples. Inhaled hypertonic saline was used to induce sputum as needed, and in case of the unavailability of expectorated sputum, suction sputum was obtained. The FAPN simultaneously detects 18 bacterial pathogens (including *S. pneumoniae, H. influenzae*, *M. catarrhalis*, *Staphylococcus aureus*, and *Pseudomonas aeruginosa*, among others) with semi-quantitative bacterial load reporting, eight viral pathogens, and seven antimicrobial resistance genes. Results are available within approximately 70 min (12).

**Fig 1:**
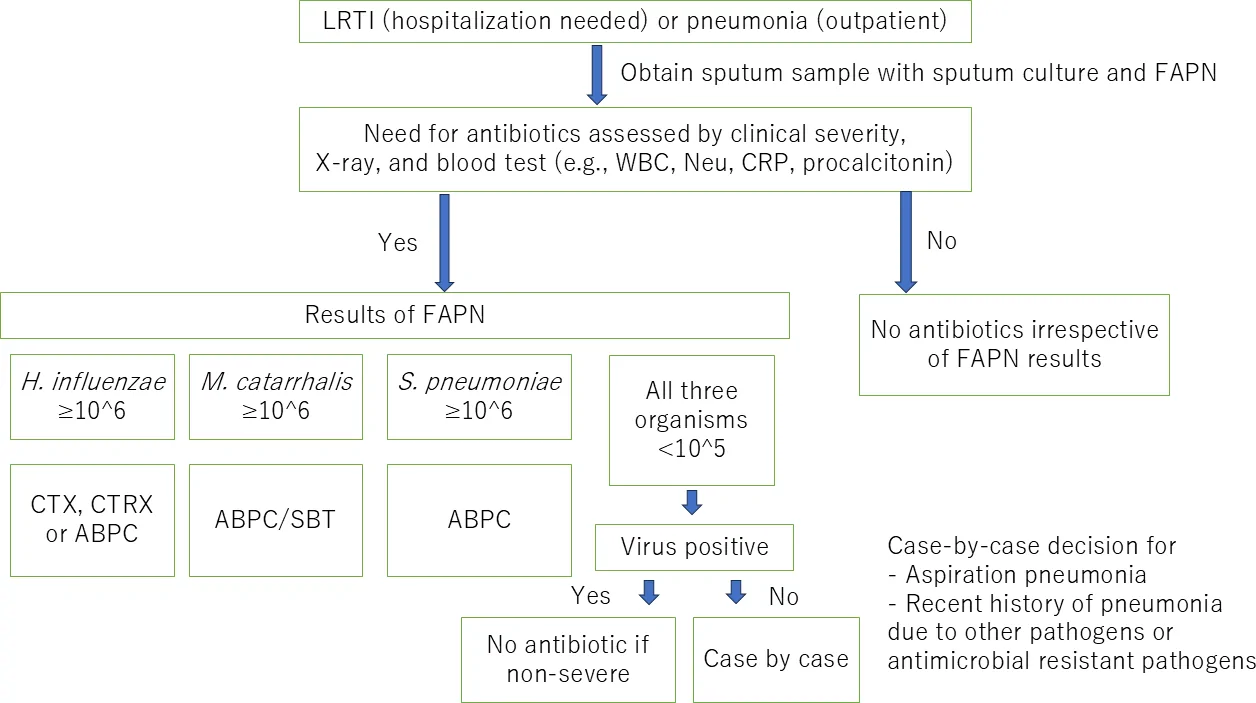
Flowchart of antimicrobial use based on FAPN results. Meeting one of the criteria warranted the need for antimicrobials. Although sputum culture and FAPN were performed in the post-FAPN period, the antimicrobial use was discouraged for cases whose antimicrobial use was not considered in the absence of FAPN. Narrow-beta lactams (e.g., ampicillin or ampicillin/sulbactam) were recommended for *S. pneumoniae* and *M. catarrhalis*, whereas clinicians considered using third-generation cephalosporins in case of *H. influenzae* due to high prevalence of beta-lactamase-negative ampicillin-resistant *H. influenzae*. Clinicians were educated that detection of organisms other than the three pathogens could often represent colonization for which antimicrobial use was not warranted. ABPC, ampicillin; ABPC/SBT, ampicillin/sulbactam; CRP, C-reactive protein; CTX, cefotaxime; CTRX, ceftriaxone; FAPN, BIOFIRE FILMARRAY Pneumonia Panel; Neu, neutrophil; WBC, white blood cell.

During the study period, all children with clinically diagnosed pneumonia and clinically diagnosed LRTI requiring hospitalization received FARP in the pre-FAPN period and FAPN in the post-FAPN period, according to the standard procedures of Nara General Medical Center. In the pre-FAPN period, antimicrobials were prescribed by physicians’ discretion, and they decided the need for antimicrobials based on patient backgrounds, clinical presentation, chest X-ray, and laboratory markers (e.g., neutrophil, C-reactive protein, procalcitonin). The hospital-wide antimicrobial stewardship team intervened in cases with longer than 7 days of broadest-spectrum antimicrobial use (i.e., vancomycin, meropenem, or equivalent antimicrobials) throughout the study period; however, none of the study participants were intervened by the hospital-wide antimicrobial stewardship team. Immediately prior to the FAPN’s introduction, all pediatricians underwent antimicrobial stewardship education with interactive discussion and developed a clinical decision flowchart guiding antimicrobial selection based on FAPN results and clinical severity (Fig. 1). This flowchart was developed to avoid unnecessary antibiotics for non-bacterial LRTIs. All pediatricians agreed to adhere to the flowchart throughout the post-FAPN period. Case-by-case decisions were made for patients with aspiration pneumonia or a recent history of pneumonia due to antimicrobial-resistant pathogens. The adherence to the flowchart for each individual patient was reconfirmed at daily department-wide conferences. In the post-FAPN period, physicians decided the need for antimicrobials and initiated antimicrobial (if needed) based on the FAPN result. All pediatricians recognized that FAPN was not a tool for initiating or broadening antibiotics in cases where they were deemed unnecessary, but rather a tool for withholding or narrowing antibiotics in cases where clinicians would otherwise initiate treatment in the absence of FAPN.

### Antimicrobial classification

Antimicrobials were classified according to the WHO AWaRe framework (6). “Access” antimicrobials included penicillins (ampicillin, ampicillin-sulbactam), first- and second-generation cephalosporins, and amoxicillin/clavulanate. “Watch” antimicrobials included third- and fourth-generation cephalosporins, carbapenems, fluoroquinolones, vancomycin, and linezolid. DOTs were calculated separately for each class among patients who received at least one agent in that class.

### Data collection

Clinical and microbiological data were retrieved by integrating electronic medical records with the Microbiology Department’s laboratory database. For each patient, the following information was collected: biological sex; age at presentation; presence of chronic comorbidities (defined as any pre-existing condition requiring regular medical follow-up); presence of concomitant bacterial infections of other systems; seasonality of presentation; multiplex panel results; concurrent culture results; and antimicrobial agents administered. Concomitant bacterial infection was defined as any bacterial infection occurring during the same clinical episode that was not attributable to bacterial pneumonia, which were detected in FAPN only, and comprised two categories: (i) infections affecting organ systems other than the lower respiratory tract (i.e., urinary tract infection, bacterial upper respiratory tract infection, meningitis, bacteremia/sepsis); and (ii) atypical respiratory pathogens (*Mycoplasma pneumoniae* and *Chlamydophila pneumoniae*) detected from respiratory specimens. Typical bacterial pathogens detected from respiratory specimens (e.g., *S. pneumoniae*, *H. influenzae*, *M. catarrhalis*) were not included in this variable because these were not well captured in the pre-FAPN period. This definition was applied consistently across both study periods. For hospitalized patients, LOS in days was calculated from admission to discharge. DOTs were calculated from medication administration records and were computed only for patients treated with at least one antimicrobial agent.

### Outcomes

Primary outcome was antimicrobial use, defined as prescription of at least one antimicrobial agent (yes/no). Secondary outcomes included (i) total DOTs among treated patients; (ii) “Access” DOTs; (iii) “Watch” DOTs; (iv) rapid pathogen identification rate (detection of ≥1 pathogen by the multiplex panel); (v) hospitalization rate; and (vi) LOS among hospitalized patients.

### Statistical analysis

Continuous variables were described as medians and IQRs. Categorical variables were described as absolute numbers and percentages. Age was categorized as <2, 2–4, 5–11, and 12–17 years. Comparisons between pre- and post-FAPN groups used the Mann–Whitney U test for continuous variables and the chi-squared or Fisher’s exact test for categorical variables, as appropriate.

Multivariable regression evaluated the independent association of FAPN implementation with each outcome. Logistic regression was used for binary outcomes (antimicrobial use, pathogen detection, hospitalization) to report odds ratios (OR). Generalized linear model (GLM) regression with log-link negative binomial distribution was used for quantitative outcomes (DOTs, LOS), reporting β coefficients due to the data overdispersion. Three nested models were fitted:

M1: unadjusted regression;

M2: adjustment for biological sex and age class;

M3: M2 with further adjustment for concomitant bacterial infections, chronic comorbidities, and seasonality.

Subgroup analyses were conducted for antimicrobial use and DOTs, stratified by hospitalization status.

In addition to the above multivariable regression models, the interrupted time-series analysis (ITSA) was performed using beta regression for antimicrobial use rate and negative binomial GLM for total, “Access,” and “Watch” DOTs, with bimesters as time units and age group, sex, seasonality, presence of comorbidity, and concomitant bacterial infection in other systems as covariates to investigate the potential time-associated impact on the outcome. Autocorrelation was addressed using Newey–West standard errors, and seasonality was accounted for in all ITSA models. Statistical significance was set at *P* < 0.05. The required sample sizes were 302 and 151 in the pre- and post-FAPN periods to detect statistical significance with 0.9 of power, a 2:1 sample size ratio, and antimicrobial use rates of 75% and 60%. All analyses were conducted using StataMP 17 (StataCorp LLC, College Station, TX, USA).

## RESULTS

### Study population

During the study period (April 2023–April 2026), 815 children were enrolled: 590 in the pre-FAPN period and 225 in the post-FAPN period. The overall median age was 2.3 years (IQR: 1.0–5.5); 440 children (54.0%) were male. Children aged under 2 years constituted the largest age group (356/815; 43.7%). Chronic comorbidities were documented in 295 patients (36.2%), and concomitant bacterial infections of other organ systems were present in 131 subjects (16.1%). Table 1 summarizes population characteristics by study period.

**TABLE 1:** Characteristics of the study population, stratified by study period (pre-FAPN vs post-FAPN)^*b*^^,^^*c*^^,^^*d*^^,^^*e*^^,^^*g*^

	Pre-FAPN panel implementation (*N* = 590)	Post-FAPN panel implementation (*N* = 225)	Total	*P*-value
Qualitative variables (as absolute numbers and percentages)				
Biological sex				
Male	315 (53.39%)	125 (55.55%)	440 (53.99%)	0.579
Female	275 (46.61%)	100 (44.45%)	375 (46.01%)	
Age class				
<2 years	244 (41.36%)	112 (49.78%)	356 (43.68%)	0.021*
2–4 years	153 (25.93%)	63 (28.00%)	216 (26.50%)	
5–11 years	152 (25.76%)	36 (16.00%)	188 (23.07%)	
12–17 years	41 (6.95%)	14 (6.22%)	55 (6.75%)	
Season of access				
December–February	84 (14.24%)	37 (16.44%)	121 (14.84%)	0.073
March–May	167 (28.31%)	81 (36.00%)	248 (30.43%)	
June–August	188 (31.86%)	63 (28.00%)	251 (30.80%)	
September–November	151 (25.59%)	44 (19.56%)	195 (23.93%)	
Concomitant bacterial infection^*a*^	96 (16.27%)	35 (15.56%)	131 (16.07%)	0.804
Urinary tract infection	4 (0.68%)	1 (0.44%)	5 (0.61%)	0.703
Bacterial upper respiratory tract infection (e.g., peritonsillar abscess and otitis media)	8 (1.36%)	5 (2.22%)	13 (1.59%)	0.378
Atypical organisms for pneumonia (i.e., *Mycoplasma pneumoniae*, *Chlamydophila pneumoniae*, *Bordetella pertussis*, and *Bordetella parapertussis*)*^e^*	102 (19.28%)	9 (4.09%)	111 (14.82%)	†^*f*^
Meningitis	1 (0.17%)	0 (0.00%)	1 (0.12%)	0.537
Bacteremia/sepsis	3 (0.51%)	2 (0.88%)	5 (0.61%)	0.534
Comorbidities	206 (34.91%)	89 (39.56%)	295 (36.20%)	0.218
Respiratory conditions	92 (15.6%)	23 (10.2%)	115 (14.11%)	
Cardiological conditions	10 (1.69%)	6 (2.67%)	16 (1.96%)	
Gastrointestinal/liver conditions	29 (4.91%)	8 (3.55%)	37 (4.54%)	
Endocrinological conditions	42 (7.12%)	19 (8.44%)	61 (7.48%)	
Allergies and autoimmune conditions	41 (6.95%)	8 (3.55%)	49 (6.01%)	
Neurological conditions	59 (10.00%)	38 (16.89%)	97 (11.90%)	
Genetic diseases and congenital malformations	41 (6.95%)	29 (12.89%)	70 (8.59%)	
Renal/urogenital conditions	23 (38.98%)	8 (3.55%)	31 (38.04%)	
Immune deficiency (primary and secondary)	2 (0.34%)	2 (0.89%)	4 (0.49%)	
Hematological conditions	11 (1.86%)	4 (1.78%)	15 (1.84%)	
Preterm birth	15 (2.54%)	15 (6.67%)	30 (3.68%)	
Antimicrobial therapy	447 (75.76%)	125 (55.55%)	572 (70.18%)	<0.001*
Ampicillin/amoxicillin	74 (12.54%)	36 (16.00%)	110 (13.50%)	
Ampicillin sulbactam/amoxicillin clavulanate	27 (4.58%)	16 (7.11%)	43 (5.28%)	
Third-generation cephalosporins	290 (49.15%)	89 (39.55%)	379 (46.50%)	
Macrolides	88 (14.91%)	8 (3.56%)	96 (11.78%)	
Tetracyclines	79 (13.39%)	2 (0.89%)	81 (9.94%)	
Carbapenems	1 (0.17%)	4 (1.78%)	5 (0.61%)	
Pathogen identification				
Positive	529 (89.66%)	220 (97.78%)	749 (91.90%)	<0.001*
Negative	61 (10.34%)	5 (2.22%)	66 (8.10%)	
Pathogen detection—number and percentage of positive samples*^b^*				
Pathogens detected by both FARP and FAPN				
HRV/Enterovirus	283 (53.5%)	121 (55.0%)	404 (53.9%)	†^*f*^
RSV	160 (30.3%)	57 (25.9%)	217 (29.0%)	†^*f*^
Parainfluenza virus	100 (18.9%)	54 (24.5%)	165 (22.0%)	†^*f*^
Adenovirus	55 (10.4%)	37 (16.8%)	92 (12.3%)	†^*f*^
hMPV	56 (10.6%)	11 (5.0%)	67 (8.9%)	†^*f*^
Coronavirus	31 (5.9%)	37 (16.8%)	72 (9.6%)	†^*f*^
Influenza virus	14 (2.6%)	17 (7.7%)	–	†^*f*^
SARS-CoV-2	19 (3.6%)	–	–	†^*f*^
*Mycoplasma pneumoniae*	101 (19.1%)	9 (4.1%)	110 (14.7%)	†^*f*^
Pathogens detected by FARP only (not included in FAPN)				
*Chlamydophila pneumoniae*	0 (0.0%)	–	–	
*Bordetella pertussis*	0 (0.0%)	–	–	
Pathogens detected by FAPN only — any detection or concentration ≥10⁵ CFU/mL				
*Moraxella catarrhalis*	–	125 (56.8%)/117 (53.2%)	–	
*Haemophilus influenzae*	–	123 (55.9%)/109 (49.5%)	–	
*Streptococcus pneumoniae*	–	101 (45.9%)/74 (33.6%)	–	
*Staphylococcus aureus*	–	54 (24.5%)/32 (14.5%)	–	
*Streptococcus pyogenes*	–	18 (8.2%)/16 (7.3%)	–	
*Pseudomonas aeruginosa*	–	12 (5.5%)/11 (5.0%)	–	
*Streptococcus agalactiae*	–	7 (3.2%)/4 (1.8%)	–	
*Serratia marcescens*	–	6 (2.7%)/5 (2.3%)	–	
*Escherichia coli*	–	7 (3.2%)/2 (0.9%)	–	
Other bacteria (*Klebsiella* spp., *Enterobacter cloacae* complex, *Acinetobacter* spp., *Proteus* spp.)	–	5 (2.3%)/1 (0.5%)	–	
Hospitalization	495 (83.90%)	178 (79.11%)	673 (82.58%)	0.107
Supplemental oxygen	298 (50.51%)	91 (40.44%)	389 (47.73%)	0.010*
High-flow nasal cannula	86 (14.58%)	33 (14.67%)	119 (14.60%)	0.974
Invasive ventilation	32 (5.24%)	23 (10.22%)	55 (6.75%)	0.015*
Quantitative variables (as medians and IQRs)				
DOTs (treated patients only)	5.67 (4.00–10.00)	5.00 (4.00–9.00)	5.33 (4.00–9.67)	<0.001*
DOTs for “Access” antimicrobials (Access-treated patients only)	5.00 (3.50–6.67)	5.00 (3.00–10.00)	5.00 (3.50–7.00)	0.040*
DOTs for “Watch” antimicrobials (Watch-treated patients only)	5.00 (3.67–8.00)	4.33 (2.00–5.00)	4.67 (3.33–7.00)	<0.001*
LOS (hospitalized patients only)	6 (5–8)	7 (6–8)	6 (5–8)	0.118

^*a*^ Concomitant bacterial infection was defined as any bacterial infection occurring during the same clinical episode that was not attributable to bacterial pneumonia which were detected in FAPN only, and comprised two categories: (i) infections affecting organ systems other than the lower respiratory tract (i.e., urinary tract infection, bacterial upper respiratory tract infection, meningitis, bacteremia/sepsis); and (ii) atypical respiratory pathogens (*Mycoplasma pneumoniae* and *Chlamydophila pneumoniae*) detected from respiratory specimens. Typical bacterial pathogens detected from respiratory specimens (e.g., *S. pneumoniae*, *H. influenzae*, *M. catarrhalis*) were not included in this variable because these were not well captured in the pre-FAPN period. This definition was applied consistently across both study periods.

^*b*^ Pathogen detection rates are expressed as percentages of samples that tested positive (pre-FAPN: *N* = 529; post-FAPN: *N* = 220). Direct comparison of individual pathogen rates between periods should be interpreted with caution, as the FARP and FAPN differ substantially in their pathogen targets and specimen types (nasopharyngeal swab vs sputum). “–” indicates that the pathogen was not included in the respective panel’s detection targets.

^*c*^ Semi-quantitative bacterial load data are available only for the post-FAPN period. “≥10⁵ CFU/mL” denotes detections at or above the threshold generally considered indicative of causative infection rather than colonization.

^*d*^ Antimicrobial resistance genes are detected by the FAPN only. mecA/C and MREJ were detected in 16 post-FAPN patients (7.1%) and CTX-M in 4 (1.8%); all other resistance genes targeted by the FAPN (IMP, KPC, NDM, OXA-48-like, VIM) were not detected. Of the 16 patients with mecA/C and MREJ detection, 12 (75.0%) received vancomycin.

^*e*^ Breakdown of atypical organisms: *Mycoplasma pneumoniae*: pre-FAPN 101 (19.09%), post-FAPN 9 (4.09%); *Chlamydophila pneumoniae*: pre-FAPN 0 (0.00%), post-FAPN 0 (0.00%); *Bordetella pertussis*: pre-FAPN 0 (0.00%), post-FAPN not assessed; and *Bordetella parapertussis*: pre-FAPN 1 (0.19%), post-FAPN not assessed. *Bordetella* spp. were not included in the FAPN panel and were therefore not assessed in the post-FAPN period.

^*f*^ Direct statistical comparison between periods is not shown for individual pathogen rates, as FARP and FAPN use different specimen types and detect different pathogen sets.

^*g*^ IQR, interquartile range; DOTs, days of therapy; LOS, length of stay; FAPN panel, BIOFIRE FILMARRAY Pneumonia Panel; LRTI, lower respiratory tract infection; * represents a statistical significance (*P*<0.05).

Antimicrobials were prescribed to 572 patients (70.2%) overall. The median total DOTs was 5.33 (IQR: 4.00–9.67); median “Watch” DOTs were 4.67 (IQR: 3.33–7.00); and median “Access” DOTs were 5.00 (IQR: 3.50–7.00) among those receiving each respective class. Rapid pathogen identification was achieved in 749 cases (91.9%). Hospitalization occurred in 673 cases (82.6%), with a median LOS of 6 days (IQR: 5–8).

### Comparison of pre-FAPN and post-FAPN periods

Post-FAPN patients were significantly younger than pre-FAPN patients (pre-FAPN median 2.9 vs post-FAPN 2.0 years; *P* < 0.001). The proportion of children under 2 years was higher in the post-FAPN period (pre-FAPN 41.4% vs post-FAPN 49.8%; *P* = 0.034), while the 5- to 11-year age group was less represented (pre-FAPN 25.8% vs post-FAPN 16.0%; *P* = 0.004). No significant differences were found in sex distribution, prevalence of comorbidities, or concomitant bacterial infections between periods.

Antimicrobial prescribing rates decreased significantly in the post-FAPN period (pre-FAPN 75.8% vs post-FAPN 55.6%; *P* < 0.001). Both “Watch” antibiotic use (pre-FAPN 59.5% vs post-FAPN 44.4%; *P* < 0.001) and “Access” antibiotic use (pre-FAPN 31.5% vs post-FAPN 23.6%; *P* = 0.040) declined. Among treated patients, median “Watch” DOTs were shorter in the post-FAPN period (pre-FAPN 5.00 [IQR: 3.67–8.00] vs post-FAPN 4.33 [2.00–5.00] days; *P* < 0.001), while “Access” DOTs were not significantly different (pre-FAPN 5.00 [3.50–6.67] vs post-FAPN 5.00 [3.00–10.00] days; *P* = 0.794).

Rapid pathogen identification improved significantly (pre-FAPN 89.7% vs post-FAPN 97.8%; *P* < 0.001). Hospitalization rates did not differ significantly in unadjusted comparison (pre-FAPN 83.9% vs post-FAPN 79.1%; *P* = 0.108). LOS among hospitalized patients did not differ significantly (pre-FAPN median 6 [IQR: 5–8] vs post-FAPN 7 [6–8] days; *P* = 0.128). The numerically longer median LOS in the post-FAPN period likely reflects the higher proportion of young infants admitted.

### Regression analysis

Results of the multivariable regression analysis are presented in Table 2, which provides details for all models. However, for briefness’s sake, the present paragraph will only present some of the results. Models not mentioned in the main text are described in detail in the aforementioned table.

**TABLE 2:** Regression analysis^*a*^^,^^*b*^

Outcome	Model	Test statistic	95% CI	*P*-value
Antimicrobial therapy	M1	OR: 0.40	0.29–0.55	<0.001*
	M2	aOR: 0.41	0.29–0.57	<0.001*
	M3	aOR: 0.39	0.28–0.55	<0.001*
Pathogen detection	M1	OR: 5.07	2.01–12.80	0.001*
	M2	aOR: 4.62	1.81–11.76	0.001*
	M3	aOR: 4.47	1.75–11.45	0.002*
Hospitalization	M1	OR: 0.73	0.49–1.07	0.108
	M2	aOR: 0.64	0.43–0.95	0.028*
	M3	aOR: 0.53	0.34–0.83	0.006*
DOTs (treated subjects only)	M1	β: −0.23	−0.44 to −0.02	0.033*
	M2	aβ: −0.25	−0.46 to −0.04	0.020*
	M3	aβ: −0.27	−0.48 to −0.05	0.017*
“Access” DOTs (treated subjects only)	M1	β: 0.04	−0.29–0.37	0.794
	M2	aβ: 0.04	−0.29–0.38	0.794
	M3	aβ: 0.06	−0.29–0.41	0.744
“Watch” DOTs (treated subjects only)	M1	β: −0.39	−0.63 to −0.15	0.002*
	M2	aβ: −0.39	−0.63 to −0.15	0.001*
	M3	aβ: −0.41	−0.66 to −0.16	0.001*
LOS (hospitalized subjects only)	M1	β: 0.10	−0.08–0.28	0.271
	M2	aβ: 0.10	−0.08–0.29	0.262
	M3	aβ: 0.09	−0.09–0.27	0.326
Subgroup analysis				
Antimicrobial therapy (non-hospitalized subjects)	M1	OR: 0.35	0.16–0.77	0.009*
	M2	aOR: 0.61	0.26–1.43	0.258
	M3	aOR: 0.44	0.16–1.16	0.096
Antimicrobial therapy (hospitalized subjects)	M1	OR: 0.40	0.28–0.57	<0.001*
	M2	aOR: 0.39	0.27–0.56	<0.001*
	M3	aOR: 0.36	0.25–0.53	<0.001*
DOTs (non-hospitalized subjects)	M1	β: 0.28	−0.18–0.73	0.233
	M2	aβ: 0.27	−0.22–0.76	0.276
	M3	aβ: 0.22	−0.32–0.75	0.428
DOTs (hospitalized subjects)	M1	β: −0.34	−0.58 to −0.10	0.006*
	M2	aβ: −0.34	−0.59 to −0.10	0.005*
	M3	aβ: −0.36	−0.61 to −0.11	0.005*

^*a*^ "*” represents a statistical significance;OR, odds ratio; aOR, adjusted odds ratio; 95% CI, 95% confidence interval; DOTs, days of therapy; LOS, length of stay; FAPN panel, BIOFIRE FILMARRAY Pneumonia Panel; M1, unadjusted regression; M2, adjustment for biological sex and age class; M3, M2 with further adjustment for concomitant bacterial infections, presence of comorbidities, and seasonality.

^*b*^ The independent variable is always represented by the systematic implementation of the FAPN panel, binomially distributed as “yes” (1) or “no” (0). Logistic regression models’ test statistic is represented by odds ratios (OR), while GLM regression was synthesized by β coefficients. Subgroup analysis is shown at the bottom of the table.

The antimicrobial use showed significantly lower odds following FAPN implementation across all models (M1: OR 0.40, 95% CI: 0.29–0.55; M3: aOR 0.39, 95% CI: 0.28–0.55; *P* < 0.001 for all). Rapid pathogen identification was significantly associated with FAPN implementation in all models (M3: aOR 4.47, 95% CI: 1.75–11.45; *P* = 0.002). Hospitalization rates, while not significantly different in the unadjusted model (M1: OR 0.73, *P* = 0.108), showed significant inverse association with FAPN implementation after adjustment (M2: aOR 0.64, *P* = 0.028; M3: aOR 0.53, 95% CI: 0.34–0.83; *P* = 0.006).

Total DOTs were significantly reduced in treated patients across all models (M3: aβ −0.27, 95% CI: −0.48 to −0.05; *P* = 0.017). Analysis by AWaRe classification revealed a selective reduction in “Watch” antibiotic DOTs (M3: aβ −0.41, 95% CI: −0.66 to −0.16; *P* = 0.001), while “Access” DOTs were not significantly affected across any model (M3: aβ 0.06, 95% CI: −0.29–0.41; *P* = 0.744). LOS did not show significant association with FAPN implementation in any model. Subgroup analyses confirmed significant reductions in antimicrobial use and DOTs among hospitalized patients, with maintained significance in fully adjusted models (aOR 0.36, *P* < 0.001; aβ −0.36, *P* = 0.005). For non-hospitalized subjects, the association with antimicrobial use was significant only in the unadjusted model and lost significance after adjustment.

### Interrupted time-series analysis

Results of the ITSA are presented in Table 3. Beta regression confirmed a significant reduction in antimicrobial prescription rates following FAPN implementation (β: −0.53; 95% CI: −0.84 to −0.21; *P* = 0.001), consistent with the multivariable regression findings. Notably, ITSA identified a significant reduction in “Watch” antibiotic DOTs (β: −0.34; 95% CI: −0.41 to −0.27; *P* ≤ 0.001).

**TABLE 3:** Interrupted time-series analysis, adjusted for age group distribution, biological sex, seasonality, comorbidities, and concomitant bacterial infections^*a*^^,^^*b*^

Outcome	β	95% CI	*P*-value
DOTs	−0.23	−0.48–0.02	0.071
“Watch” DOTs	−0.34	−0.41 to −0.27	<0.001*
“Access” DOTs	−0.09	−1.49 to −1.32	0.905
Antimicrobial therapy usage rate	−0.53	−0.84 to −0.21	0.001*
“Watch” usage rate	0.45	−0.50–1.40	0.352
“Access” usage rate	−0.11	−1.05–0.83	0.815

^*a*^ 95% CI, 95% confidence interval; DOTs, days of therapy; FAPN panel, BIOFIRE FILMARRAY Pneumonia Panel; * represents a statistical significance (*P*<0.05).

^*b*^ The independent variable is always represented by the systematic implementation of the FAPN panel, binomially distributed as “yes” (1) or “no” (0). Beta regression was implemented for qualitative variables. Log-link negative binomial generalized linear model regression was used for quantitative variables with binomial distribution. All models were adjusted for seasonal distribution and autocorrelation; the Newey-West standard errors were employed for the latter. For the “Watch” usage rate beta-regression model, a “1” value was replaced with 0.999999 for statistical purposes.

## DISCUSSION

This retrospective cohort study evaluated the clinical impact of the systematic introduction of the FAPN, combined with the interactive antimicrobial stewardship education, in pediatric patients with LRTIs at a tertiary center in Japan. The principal findings were: (i) a significant reduction in antimicrobial prescribing rates; (ii) a selective reduction in “Watch” antibiotic DOTs, with no significant change in “Access” DOTs—indicating a shift toward more targeted, guideline-appropriate prescribing; and (iii) a significant improvement in rapid pathogen identification rates. These results were largely confirmed by ITSA.

The reduction in overall antimicrobial prescribing (aOR: 0.39 in fully adjusted logistic regression) is consistent with evidence from adult randomized trials demonstrating that multiplex PCR-based pneumonia diagnostics can safely facilitate the use of narrower antibiotics (20, 21). The structured stewardship education program provided at our center likely amplified this effect: the Yoshida et al. PICU study found no significant reduction in antimicrobial use without a concurrent stewardship component (17), highlighting that diagnostic tools alone are insufficient without an aligned clinical decision framework. The large reduction of antimicrobial prescription following FAPN introduction may have been associated with our strong stewardship program, which was characterized by interactive discussions by all pediatricians in the study center to develop the unanimously agreed flowchart. This led to their active and consistent involvements to follow the flowchart. To implement a new diagnostic tool, education of healthcare providers can be crucial to maximize its beneficial impacts.

The AWaRe-stratified analysis represents a key contribution of this study. The selective reduction in “Watch” antibiotic DOTs (aβ −0.41; *P* = 0.001)—without a corresponding increase in “Access” DOTs—suggests that the FAPN guided clinicians toward earlier de-escalation or discontinuation of broader-spectrum agents, while maintaining appropriate use of first-line therapies. This pattern is particularly relevant from a stewardship perspective: it implies that the intervention did not simply shift the burden from one antibiotic class to another, but genuinely reduced unnecessary broad-spectrum exposure. The ITSA findings further support this interpretation, identifying a significant reduction in “Watch” DOTs over time, possibly reflecting a progressive consolidation of stewardship practices following the FAPN’s introduction.

At the same time, the inclusion of new pathogens in the FAPN panel may have contributed to more precise identification of the circulating microbial ecosystem. The number of isolates positive for *M. catarrhalis* and *H. influenzae* (Table 1) suggests that a significant quota of bacterial infections may have been sustained by these pathogens before the panel’s implementation as well. Conversely, a cautionary interpretation is required to avoid unnecessary antimicrobial use given that all detected pathogens in the FAPN may not reflect causal pathogens. In Japan, the prevalence of beta-lactamase-negative ampicillin-resistant (BLNAR) *H. influenzae* is high for which clinicians often use third-generation cephalosporins for suspected bacterial pneumonia with unknown pathogen or unknown antimicrobial sensitivity. A Japanese study indicated that approximately 60% of *H. influenzae* was a BLNAR isolate (22). Once FAPN provided the bacterial pathogen other than *H. influenzae* (e.g., *S. pneumoniae* and *M. catarrhalis*), clinicians could avoid using the third-generation cephalosporins and start narrow-spectrum antimicrobials (e.g., ampicillin) from the beginning. This could explain why the FAPN was associated with a larger reduction in “Watch” DOTs. The semi-quantification of bacterial load integrated into the decision flowchart provided clinicians with a practical tool to distinguish likely causative pathogens from probable colonizers. Specifically, higher bacterial loads (equivalent to ≥10⁶ CFU/mL) were interpreted as more consistent with causative infection, whereas lower semi-quantitative signals—particularly for organisms known to frequently colonize the pediatric airway such as *M. catarrhalis* and *H. influenzae*—were weighed alongside clinical parameters including fever, CRP, and radiographic findings before initiating antimicrobial therapy. Conversely, the antimicrobial resistance genes targeted by the FAPN were of limited utility in guiding the management of pediatric community-acquired pneumonia in Japan, especially because BLNAR-associated genes were not included in the current FAPN panel.

Statistically significant reductions in antimicrobial use were not observed in non-hospitalized cases in the adjusted models. This could be due to a lack of power resulting from the small sample size of non-hospitalized patients with pneumonia in this study. Another possibility is that non-hospitalized cases were less likely to receive antimicrobials even without FAPN. These findings have implications for which pneumonia cases benefit most from the use of FAPN. For example, non-hospitalized cases or cases in which a clinician considers not using any antimicrobials or starting narrow-spectrum antimicrobials (e.g., ampicillin or amoxicillin) without FAPN may not receive large benefits. In other words, FAPN is more likely to benefit patients whose physicians consider starting relatively broad-spectrum antimicrobials because of unknown causal pathogens.

The significant reduction in hospitalization rates in adjusted analyses (aOR: 0.53; *P* = 0.006), despite a non-significant unadjusted difference, suggests that confounders—particularly the higher proportion of younger and potentially more vulnerable infants in the post-FAPN period—attenuated the apparent effect. The FAPN’s ability to rapidly identify or exclude bacterial pathogens may have enabled more confident early discharge decisions for patients with viral LRTIs, consistent with the findings of Yoshida et al. in a PICU context (17).

LOS did not show significant reduction in either regression analysis or ITSA. This finding may reflect the clinical context of pneumonia, which often requires a minimum duration of hospitalization for respiratory monitoring and oxygen support regardless of pathogen identification status. The randomized trial by Markussen et al. similarly found no significant reduction in LOS following syndromic PCR implementation for community-acquired pneumonia (23). These findings indicate that further investigations are required to evaluate the cost-effectiveness of this rapid molecular test, including analyses to identify which specific populations benefit most cost-effectively from the use of FAPN.

This study has several limitations. Its retrospective, single-center design limits generalizability. The introduction of the FAPN and stewardship education as a joint intervention prevents attributing changes to either component alone. Without a concomitant stewardship program, the observed findings in the post-FAPN period may not have been evident. The pre- and post-FAPN cohorts differ in age distribution—with a higher proportion of young infants post-FAPN—which may reflect secular trends rather than intervention effects; however, all regression models adjusted for age class. A large difference in the outbreak patterns between the pre- and post-FAPN was observed (i.e., Mycoplasma). Although we considered this in the multivariable models, a possibility of residual confounders should be considered. Finally, antibiotic appropriateness beyond the AWaRe framework (e.g., whether treatment was indicated at all) could not be assessed from the available data.

The strengths of this study include the structured nature of the stewardship intervention, the AWaRe-stratified antibiotic analysis—which adds granularity beyond overall antibiotic rates—and the use of ITSA to account for temporal trends and autocorrelation. To our knowledge, this is the first study to evaluate the FAPN’s impact on AWaRe-classified antibiotic use in a general pediatric ward in Japan.

In conclusion, the systematic implementation of the FAPN combined with interactive antimicrobial stewardship education was significantly associated with reduced antimicrobial prescribing, a selective reduction in “Watch” antibiotic use, improved pathogen identification, and lower hospitalization rates in pediatric patients with LRTIs. These findings support the clinical value of rapid multiplex PCR diagnostics, especially when integrated with a strong antimicrobial stewardship program in pediatric care.

## Data Availability

The authors agree to sharing relevant information in aggregated and anonymized form upon reasonable request.

## References

[1] Williams BG, Gouws E, Boschi-Pinto C, Bryce J, Dye C. 2002. Estimates of world-wide distribution of child deaths from acute respiratory infections. Lancet Infect Dis 2:25–32. doi:10.1016/s1473-3099(01)00170-0 11892493

[2] Rudan I, Boschi-Pinto C, Biloglav Z, Mulholland K, Campbell H. 2008. Epidemiology and etiology of childhood pneumonia. Bull World Health Organ 86:408–416. doi:10.2471/blt.07.048769 PMC2647437 18545744

[3] Jain S, Williams DJ, Arnold SR, Ampofo K, Bramley AM, Reed C, Stockmann C, Anderson EJ, Grijalva CG, Self WH, et al.. 2015. Community-acquired pneumonia requiring hospitalization among U.S. children. N Engl J Med 372:835–845. doi:10.1056/NEJMoa1405870 PMC4697461 25714161

[4] Bamba M, Jozaki K, Sugaya N, Tamai S, Ishihara J, Kori T, Shiro H, Takeuchi Y, Cho H, Nakao A, Okano Y, Kimura K, Komiyama O, Nonoyama M, Kobayashi I, Kato T, Sunakawa K. 2006. Prospective surveillance for atypical pathogens in children with community-acquired pneumonia in Japan. J Infect Chemother 12:36–41. doi:10.1007/s10156-005-0422-y 16506088

[5] McCullers JA. 2014. The co-pathogenesis of influenza viruses with bacteria in the lung. Nat Rev Microbiol 12:252–262. doi:10.1038/nrmicro3231 24590244

[6] World Health Organization. 2022. The WHO AWaRe (access, watch, reserve) antibiotic book. Geneva WHO. https://www.who.int/publications/i/item/9789240062382.

[7] Klugman KP, Lonks JR. 2005. Hidden epidemic of macrolide-resistant pneumococci. Emerg Infect Dis 11:802–807. doi:10.3201/eid1106.050116 PMC3367596 15963272

[8] Sulis G, Sayood S, Katukoori S, Bollam N, George I, Yaeger LH, Chavez MA, Tetteh E, Yarrabelli S, Pulcini C, Harbarth S, Mertz D, Sharland M, Moja L, Huttner B, Gandra S. 2022. Exposure to World Health Organization’s AWaRe antibiotics and isolation of multidrug resistant bacteria: a systematic review and meta-analysis. Clin Microbiol Infect 28:1193–1202. doi:10.1016/j.cmi.2022.03.014 35339675

[9] Hammitt LL, Murdoch DR, Scott JAG, Driscoll A, Karron RA, Levine OS, O’Brien KL. 2012. Specimen collection for the diagnosis of pediatric pneumonia. Clin Infect Dis 54:S132–S139. doi:10.1093/cid/cir1068 PMC3693496 22403227

[10] Yang Y, Zhu X, Sun Y, Qian K, Liu Z. 2022. Comparison of next-generation sequencing with traditional methods for pathogen detection in cases of lower respiratory tract infection at a community hospital in Eastern China. Medicine (Baltimore) 101:e32423. doi:10.1097/MD.0000000000032423 PMC9794229 36595873

[11] Blaschke AJ, Heyrend C, Byington CL, Fisher MA, Barker E, Garrone NF, Thatcher SA, Pavia AT, Barney T, Alger GD, Daly JA, Ririe KM, Ota I, Poritz MA. 2012. Rapid identification of pathogens from positive blood cultures by multiplex polymerase chain reaction using the FilmArray system. Diagn Microbiol Infect Dis 74:349–355. doi:10.1016/j.diagmicrobio.2012.08.013 PMC3496814 22999332

[12] Murphy CN, Fowler R, Balada-Llasat JM, Carroll A, Stone H, Akerele O, Buchan B, Windham S, Hopp A, Ronen S, et al.. 2020. Multicenter evaluation of the BioFire FilmArray pneumonia/pneumonia plus panel for detection and quantification of agents of lower respiratory tract infection. J Clin Microbiol 58:e00128-20. doi:10.1128/JCM.00128-20 PMC7315029 32350043

[13] Rand KH, Beal SG, Cherabuddi K, Couturier B, Lingenfelter B, Rindlisbacher C, Jones J, Houck HJ, Lessard KJ, Tremblay EE. 2021. Performance of a semiquantitative multiplex bacterial and viral PCR panel compared with standard microbiological laboratory results: 396 patients studied with the Biofire Pneumonia Panel. Open Forum Infect Dis 8:ofaa560. doi:10.1093/ofid/ofaa560 PMC7793460 33447631

[14] Peiffer-Smadja N, Bouadma L, Mathy V, Allouche K, Patrier J, Reboul M, Montravers P, Timsit J-F, Armand-Lefevre L. 2020. Performance and impact of a multiplex PCR in ICU patients with ventilator-associated pneumonia or ventilated hospital-acquired pneumonia. Crit Care 24:366. doi:10.1186/s13054-020-03067-2 PMC7303941 32560662

[15] Debbagh F, Ben Houmich T, Guennouni M, Sahraoui H, Hanchi AL, Mouaffak Y, Younous S, Soraa N. 2023. Evaluation PCR panel of the FilmArray pneumonia plus for pathogen detection of ventilator-associated pneumonia in children and its impact on therapeutic management. Iran J Microbiol 15:19–26. doi:10.18502/ijm.v15i1.11914 PMC10105283 37069910

[16] Gadsby NJ, Russell CD, McHugh MP, Mark H, Conway Morris A, Laurenson IF, Hill AT, Templeton KE. 2016. Comprehensive molecular testing for respiratory pathogens in community-acquired pneumonia. Clin Infect Dis 62:817–823. doi:10.1093/cid/civ1214 PMC4787606 26747825

[17] Yoshida K, Hatachi T, Okamoto Y, Aoki Y, Kyogoku M, Moon Miyashita K, Inata Y, Shimizu Y, Fujiwara F, Takeuchi M. 2021. Application of multiplex polymerase chain reaction for pathogen identification and antibiotic use in children with respiratory infections in a PICU. Pediatr Crit Care Med 22:e644–e648. doi:10.1097/PCC.0000000000002794 34224509

[18] Bradley JS, Byington CL, Shah SS, Alverson B, Carter ER, Harrison C, Kaplan SL, Mace SE, McCracken GH Jr, Moore MR, St Peter SD, Stockwell JA, Swanson JT. 2011. The management of community-acquired pneumonia in infants and children older than 3 months of age: clinical practice guidelines by the Pediatric Infectious Diseases Society and the Infectious Diseases Society of America. Clin Infect Dis 53:e25–e76. doi:10.1093/cid/cir531 PMC7107838 21880587

[19] Biscevic-Tokic J, Tokic N, Musanovic A. 2013. Pneumonia as the most common lower respiratory tract infection. Med Arch 67:442–445. doi:10.5455/medarh.2013.67.442-445 PMC4272454 25568518

[20] Poole S, Tanner AR, Naidu VV, Borca F, Phan H, Saeed K, Grocott MPW, Dushianthan A, Moyses H, Clark TW. 2022. Molecular point-of-care testing for lower respiratory tract pathogens improves safe antibiotic de-escalation in patients with pneumonia in the ICU: results of a randomised controlled trial. J Infect 85:625–633. doi:10.1016/j.jinf.2022.09.003 36096312

[21] Markussen DL, Serigstad S, Ritz C, Knoop ST, Ebbesen MH, Faurholt-Jepsen D, Heggelund L, van Werkhoven CH, Clark TW, Bjørneklett RO, Kommedal Ø, Ulvestad E, Grewal HMS. 2024. Diagnostic stewardship in community-acquired pneumonia with syndromic molecular testing: a randomized clinical trial. JAMA Netw Open 7:e240830. doi:10.1001/jamanetworkopen.2024.0830 PMC10918504 38446481

[22] Honda H, Sato T, Shinagawa M, Fukushima Y, Nakajima C, Suzuki Y, Shiraishi T, Kuronuma K, Takahashi S, Takahashi H, Yokota S-I. 2018. Multiclonal expansion and high prevalence of β-lactamase-negative *Haemophilus influenzae* with high-level ampicillin resistance in Japan and susceptibility to quinolones. Antimicrob Agents Chemother 62:e00851-18. doi:10.1128/AAC.00851-18 PMC6125502 29987153

[23] Brigadoi G, Gres E, Barbieri E, Liberati C, Rossin S, Chiusaroli L, Demarin GC, Tesser F, Maestri L, Tirelli F, Carrara E, Tacconelli E, Bressan S, Giaquinto C, Da Dalt L, Donà D. 2024. Impact of a multifaceted antibiotic stewardship programme in a paediatric acute care unit over 8 years. JAC Antimicrob Resist 6:dlae181. doi:10.1093/jacamr/dlae181 PMC11538966 39507942

